# Safety and Efficacy of Jenacid® Herbal Product Against Indomethacin-Induced Ulcers in Albino Wistar Rats

**DOI:** 10.7759/cureus.72606

**Published:** 2024-10-29

**Authors:** Joshua Kiprotich, Neeza Timothy, Tadele M Yadesa, Daniel C Mwandah

**Affiliations:** 1 Department of Pharmacology and Toxicology, Kampala International University Western Campus, Bushenyi, UGA; 2 Department of Clinical Pharmacy and Pharmacy Practice, Kampala International University Western Campus, Bushenyi, UGA; 3 Department of Pharmacy, Mbarara University of Science and Technology, Mbarara, UGA; 4 Department of Pharmacology and Therapeutics, Mbarara University of Science and Technology, Mbarara, UGA

**Keywords:** acute toxicity, jenacid herbal product, peptic ulcer disease, phytochemical composition, subacute toxicity

## Abstract

Background: Jenacid Herbal Product (JHP) used for treating peptic ulcer disease in Uganda, sold over the counter, is approved by the National Drug Authority as a Traditional Herbal Product number THP 482. There have been no published studies on its safety and efficacy.

Objective: This study aimed to assess potential acute and subacute toxicity as well as the efficacy of JHP.

Method: An acute toxicity test was performed on Swiss Albino Wistar rats using Lorke’s method at single oral doses of 10-5000 mg/kg. General change in behavior, adverse effects, and mortality were determined. In the subacute study, Wistar rats received daily oral doses of 250, 500, and 1000 mg/kg of JHP for 28 days. Body weight and biochemical and hematological parameters were measured at the end of the experiment. For the efficacy study, rats were randomly grouped into six groups (n=5). Gastric ulceration induced with a single oral dose of indomethacin 25 mg/kg. One group was humanely sacrificed under halothane anesthesia to confirm ulceration. Treatments included distilled water (negative control), 20 mg/kg omeprazole (positive control), 250, 500, and 1000 mg/kg of JHP for seven days, after which animals were sacrificed, stomachs excised out, observed for gastric lesions (number and severity), and scored.

Results: The acute toxicity study showed oral LD50 of JHP was above 5000 mg/kg. A subacute toxicity study of JHP had no observable toxicity symptoms or significant variation in body weight, food and water consumption, hematological parameters, or mortality. Elevated aspartate aminotransferase (AST) and alanine aminotransferase (ALT) were noted at 1000 mg/kg but within normal ranges. A significant decrease in total protein was observed at 500 mg/kg and 1000 mg/kg. JHP demonstrated ulcer-healing properties, with the highest efficacy at 500 mg/kg.

Conclusion: JHP is effective against ulcers with no significant adverse effects. However, chronic use of very high doses may cause a reduction in total protein levels.

## Introduction

Peptic ulcer disease (PUD) is a condition in which the gastrointestinal (GI) tract’s inner lining breaks down due to excessive gastric acid or pepsin effect [1]. It continues to be a major source of morbidity, hence increasing healthcare costs. PUD can manifest itself in a variety of ways, from mild cases that heal on their own to severe cases with serious complications, such as gastrointestinal bleeding and perforation, with the potential for morbidity and mortality. Peptic ulcer disease affects up to 10% of the world population [2]. A 2014-2015 survey found that 11.4% of people in Mbarara, Uganda, had PUD [2]. PUD can lead to life-threatening complications such as anemia, GI perforation, and infection if not treated. The disease primarily affects adults and is less common in young children. Helicobacter pylori infection, acetylsalicylic acid (ASA) use, and other non-steroidal anti-inflammatory drugs (NSAIDs) are the three most common contributory factors to PUD [3]. In recent years, the treatment of H. pylori infection has improved, but the use of ASA and other NSAIDs has increased simultaneously [3].

According to Brzozowska et al., the treatment of this condition requires a combination of various molecules with specific mechanisms of action [4]. Various modern medications, including prostaglandin analogs, proton pump inhibitors (PPIs), cytoprotective agents, and histamine receptor antagonists, have been applied in treating PUD [3]. However, effective treatment using these conventional medicines is not usually attained because they are associated with relapse of disease, the drugs are costly, and have several drug interactions and side effects like galactorrhea, gynecomastia, GI infections, and impotence [5-6]. As a result, the majority of people are looking for herbal products to use as therapeutic agents for a variety of ailments. Mostly, herbal remedies are thought to be safe unless a reasonable risk has been identified in humans. On the contrary, a rising number of case reports indicate that their use has resulted in acute or chronic intoxication [7]. These toxic effects range from mild gastrointestinal symptoms and allergic reactions to renal and/or hepatic toxicity, carcinogenic effects, hematological, neurological, and cardiovascular complications, and death, depending on acute or chronic consumption and the quantity consumed [7]. Only acute and severe side effects are likely to be discovered without further study. Notably, the absence of obvious hazardous consequences does not imply complete safety [8].

In Uganda, herbal medicines are often used in many more instances because they are easily accessible and widely advertised. Unlike pharmaceuticals, the purity and potency of herbal products remain unregulated [9]. They are considered "food integrators" and are available without a prescription on the market [9]. This poses a significant risk because various studies and experts have shown their potential side effects if taken irregularly, in excess quantities, or in conjunction with certain medicines [9]. For instance, when taken with conventional drugs, there can be drug interactions. Thus, this study aimed to assess the safety and anti-ulcer activity of JHP on indomethacin-induced ulcer models.

## Materials and methods

Methodology

Jena Herbal Product was obtained from Jena Herbals Limited, a recognized distributor in Mbarara, South Western Uganda. The product was verified through the inspection of labels and other physical elements. The company ensures appropriate storage methods; thus, a better product is achieved, and there is no chance of a counterfeit. The product contains extracts of two medicinal plants, Zanthoxylum chalybeum and Warburgia ugandensis, which are formulated in honey and sugar bases. The recommended dose for adults is 20 ml taken orally thrice daily for two to four weeks. Its Uganda National Drug Authority notification number is THA 482.

Study animals

Albino Wistar rats eight weeks old weighing 150-220 g of either sex were used for this experiment. These were obtained from the KIU-WC pharmacology animal house. They were kept in normal cages and subjected to regular husbandry settings, such as relative humidity of 55±10%, a temperature of 23±2 °C, and a 12-hour light-dark cycle. The animals were given clean water and food pellets throughout the trial. The animals were given two weeks to acclimate before the trial. Animal procedures were carried out in compliance with the National Institutes of Health's guidelines for Human Care and Use of Laboratory Animals (OECD, 2000) as well as WHO guidelines (2004) [10]. The experimental protocol was approved by the Institutional Animal Ethical Committee (IAEC), protocol number SOPRC/002/24.

Study design and setting

This study was an in vivo experiment that evaluated JHP's acute toxicity, sub-acute toxicity, and anti-ulcer activity. A preliminary phytochemical analysis was also done on the product. One set of laboratory rats was used as a control group, and the others were used as experimental groups. The experiments occurred in pharmacology and pharmacognosy laboratories at Kampala International University-Western campus, School of Pharmacy, and the pharmaceutical analysis laboratory at Mbarara University of Science and Technology.

Extract preparation and storage

The herbal product was taken to the pharmaceutical analysis laboratory at Mbarara University of Science and Technology and concentrated using the freeze-drying method [11]. To maintain the quality of phytochemicals or other components included in the preparation, the liquid was first deep frozen at −800 °C, placed into a freeze tray, and the solvent was blown off for 8-12 hours using a blow vacuum concentrator/evaporator model till semi-solid extract was obtained [11]. The extract collected was kept in the refrigerator between 2 and 80 °C.

During phytochemical analysis and animal studies, the extract was reconstituted to a concentration of 400 mg/ml using distilled water. Several doses in mg/kg of body weight, as well as the volume to give, were determined using the Ghosh 1984 formula before administration.

$Volume to administer = (dose(mg/kg) &times; weight(kg))/concentration(mg/ml)$

Preliminary Phytochemical Screening

Standard procedures were used to test the product for secondary metabolites such as flavonoids, tannins, saponins, glycosides, terpenoids, alkaloids, steroids, and anthraquinones in order to link the anti-ulcer action of JHP to the presence or absence of these active ingredients [12].

Test for alkaloids (Wagner’s test): In a test tube with 2 ml of product, a few drops of Wagner's reagent were added (dilute iodine solution). The formation of a reddish-brown precipitate suggested the presence of alkaloids [12].

Test for saponins (foam test): Two milliliters of the product were mixed with 5 ml water and shaken for a few minutes. Saponins were detected in the form of froth that lasted 60-120 seconds [12].

Test for tannins (ferric chloride test): In a test tube, a few drops of 5% ferric chloride solution were added to 3 ml of aqueous product. The formation of a deep blue-black tint suggested the presence of tannins [12].

Test for coumarins: Three drops of alcoholic iron (iii) chloride solution, followed by concentrated nitric acid, were added to the concentrated sample in a test tube. Coumarins were detected by a vivid green tint that turned yellow [12].

Test for anthraquinones (Bontrager’s test): One gram of the product was mixed with 7 ml of weak hydrochloric acid and heated for 10 minutes in a water bath before being filtered and refrigerated. After extracting the filtrate with carbon tetrachloride, it was mixed with an equal volume of ammonia solution and shaken. The formation of a red tint in the ammoniacal layer indicated the presence of an anthraquinone moiety [12].

Test for flavonoids: One milliliter of ethanol and four drops of 2% lead acetate were added to 2 ml of the product. The formation of an orange precipitate indicated the presence of flavonoids [12].

Test for essential oils: Three milliliters of the product in a test tube were shaken with 0.2 ml of 2 M sodium hydroxide solutions, followed by 2 ml of hydrochloric acid. A white precipitate was observed, indicating the absence of essential oils [12].

Test for sterols and triterpenoids (Liebermann-Burchard’s test): Two milliliters of the product were gently combined with several drops of acetic anhydride and 2 ml of strong sulfuric acid. The absence of sterols and triterpenoids was indicated by no apparent alterations [12].

Test for reducing sugars: In a test tube with 2 ml of product, Benedict solution was added and heated for three to five minutes in a water bath [12].

Experimental Design for Acute Toxicity (14 Day)

The oral median lethal doses (LD50) of JHP were estimated according to Lorke’s method [13]. A total of 12 Wistar female rats were randomly allocated into two phases.

In phase I, nine healthy rats were randomly divided into three groups (n=3) and dosed via the oral route. Each group was administered different doses of 10 mg/kg, 100 mg/kg, and 1000 mg/kg body weight of JHP to groups I, II, and III, respectively. Each of the treated rats was observed continuously for the first four hours for behavioral, autonomic, and neurologic profile and manifestation of physical signs of toxicity such as loss of appetite and scored for mortality after 24 hours.

In phase II, three healthy rats were used; they were randomly distributed into three groups (n=1). Higher doses of JHP (1600, 2900, and 5000 mg/kg body weight) were administered sequentially to groups I, II, and III, respectively, via oral route and individual animals were observed for neurologic profiles (gait, touch response, reactivity, pain response and spontaneous activity), behavioral profiles (restlessness, fearfulness, alertness, and irritability), autonomic profiles (urination and defecation), and physical states such as salivation, tremors, lacrimation, hair erection, loss of appetite, diarrhea, and morbidity or mortality, for two hours continuously and periodically during the first 24 [13] and observed then once a day for 14 days. The geometric mean of the maximum dose causing 0% death and the minimum dose causing 100% mortality was used to compute the LD50:

$LD50 = &radic;(D0&times; D100)$

$D100 = lowest dose that gave no mortality$

$D0 = Highest dose that gave mortality$

Experimental Design for Subacute Toxicity (28 days)

The subacute toxicity of JHP was determined using the OECD method.

Treatments: Four experimental groups (n=5) were designed for the evaluation of subacute toxicity: control (given orally distilled water for 28 days) and test (treated by oral gavage for 28 days with different dosages of the JHP: 250, 500, or 1000 mg/kg body weight). The dosages for the subacute toxicity test were estimated using LD50 and the OECD Guideline 407 dose calculation. During the 28-day treatments, the animals were checked daily for general health and clinical toxicity signs, and body weight changes were recorded on days 1, 7, 14, 21, and 28. At the end of the trial period, all animals were fasted overnight before blood sampling. Under ether anesthesia, blood samples were obtained from the abdominal aorta in two types of tubes: one with EDTA and the other without. Hematological parameters were immediately determined in the tube with EDTA. The serum for biochemical analysis was obtained by centrifuging the second tube at 3000 rpm for 10 minutes at 4 °C.

Hematological analysis

An automatic hematological analyzer was used to do the hematological examination. White blood cells (WBC), granulocytes (GRA), lymphocytes (LYM), mean corpuscular hemoglobin (MCH), hemoglobin (HGB), mean platelet volume (MPV), red blood cells (RBC), mean corpuscular volume (MCV), red cell distribution width (RDW), mean corpuscular hemoglobin concentration (MCHC), platelets (PLT), and hematocrit (HCT) were the hematological parameters measured.

Biochemical analysis

A semi-automatic biochemical analyzer was used to evaluate serum samples for biochemical parameters such as liver function parameters (alanine aminotransferase [ALT], aspartate aminotransferase [AST], and total protein), renal function profile, and other factor parameters at the KIU biochemistry laboratory.

Evaluation of Anti-Ulcer Activity of JHP

Thirty animals of both sexes (weighing 100-250 g, 8-12 weeks old) were used for this study. These were randomly grouped into six groups of five each. The animals were denied food and water and observed for five hours. Gastric ulceration was induced in all groups by giving a single oral dose of indomethacin at 25 mg/kg body weight [14]. Five hours after ulcer induction, group VI was humanely sacrificed under halothane anesthesia, and the stomach was excised out and opened along greater curvature to confirm the ulceration. Group II (positive control) received 20 mg/kg omeprazole orally, group III, IV, and V received 250 mg/kg, 500 mg/kg, and 1000 mg/kg of JHP orally, respectively, while group I (negative control) received distilled water. All groups of animals received their treatments twice daily for seven days.

On the eighth day, groups II, III, IV, V, and I were anesthetized with ether and then sacrificed. The abdominal cavities of the animals were opened, and the stomachs were excised; stomachs were then opened along the greater curvature, cleaned with distilled water to clearly observe the gastric lesions (number and severity), and scored [8] (0 = no ulcer, 0.5 = red coloration, 1 = spot ulcer, 2 = number of ulcers less than 5, 3 = number of ulcers more than or equal to 5, 4 = ulcer with bleeding, and 5 = perforation of gastric/duodenal wall) [8]. The ulcer index was then calculated.

Ulcer index (UI) = Severity of ulcer + Total number of ulcers. 

Healing index (%) = \((UI_control_ - UI_pretreated_) × 100/UI_control_\)

Statistical analysis

The data were analyzed using the Statistical Package for Social Sciences (SPSS) software version 21 (IBM Corp., Armonk, NY). Group means were determined and compared using analysis of variance (ANOVA) with the least significant difference (LSD post hoc test). At P<0.05, differences were considered significant.

## Results

Phytochemical screening

As shown in Table 1, preliminary phytochemical screening of Jenacid® herbal product showed the existence of many secondary metabolites.

**Table 1:** Phytochemical constituents of Jenacid® herbal product A detailed screening of secondary metabolites indicates the presence or absence of alkaloids, saponins, flavonoids, and other active compounds responsible for the potential anti-ulcer activity. −: absence; +: presence.

S/no.	Chemical constituents	Tests performed	Results
1	Alkaloids	Wagner’s test	+
2	Saponins	Froth test	+
3	Tannins	Ferric chloride test	+
4	Coumarins	Coumarin test	+
5	Anthraquinones	Bontrager’s test	−
6	Flavonoids	Flavonoid test	+
7	Essential oils	Essential oil test	−
8	Sterols and triterpenoids	Liebermann-Burchard’s test	+

Acute toxicity study

Within the first 24 hours and over the 14-day follow-up, no mortality or changes in behavioral, autonomic, neurologic, or physical profiles were seen in the acute toxicity test at the limit test dose of 5000 mg/kg, indicating that the LD50 of Jenacid® Herbal Product is above 5000 mg/kg/day body weight. This indicates the product is practically not harmful at doses up to the limit dose tested.

Sub-acute Toxicity Study

Body weight, water, and dietary intake: The subacute administration of Jenacid® Herbal Product did not cause any evident toxicity or mortality in any of the treated rats at all doses utilized. Furthermore, water and food consumption in rats treated subacutely with repeated oral doses of the product (250, 500, or 1000 mg/kg) showed no significant changes. Throughout the 28-day trial period, both the treated rats and the control appeared to be in good health. Table 2 shows that during the entire experiment period, there were insignificant differences in the weights of treated groups with doses up to 1000 mg/kg compared to the control group (P < 0.05).

**Table 2:** Effect of subacute oral administration of Jenacid® on body weight in albino Wistar rats Displays the changes in body weight of rats over a 28-day period, demonstrating the safety profile of Jenacid® across different doses. Note: Each value represents the mean ± SEM for each group (n=5).

Parameter	G 1(JH_250_)	G 2(JH_500_)	G 3(JH_1000_)	G4(Control)
1st weights	159.340 ± 4.80	179.080 ± 2.49	165.560 ± 3.41	146.080 ± 3.14
2nd weights	172.000 ± 7.09	188.740 ± 4.55	177.940 ± 5.44	172.840 ± 4.58
3rd weights	165.320 ± 7.44	178.760 ± 2.54	170.940 ± 3.75	176.900 ± 5.64
4th weights	176.740 ± 8.52	194.260 ± 4.77	185.020 ± 3.73	174.320 ± 5.27

Hematological parameters

Hematological parameters, which included MPV, HGB, RBC, HCT, WBC, MCH, MCHC, MCV, and RDW in rats treated with Jenacid® Herbal Product (250, 500, and 1000 mg/kg), show an insignificant difference from the control rats (Table 3) (P < 0.05).

**Table 3:** Hematological parameters in rats following subacute administration of Jenacid® Compares key blood parameters (e.g., hemoglobin, red blood cell count, white blood cell count) to assess any hematological toxicity after repeated doses of Jenacid®. Note: Each value represents the mean ± SEM for each group (n=5). HGB: hemoglobin, RBC: red blood cells, HCT: hematocrit, RDW: red cell distribution width, WBC: white blood cells, PLT: platelets.

Parameter	G 1(JH_250_)	G 2(JH_500_)	G 3(JH_1000_)	G4(Control)
HGB (g/dL)	15.400 ± 0.45	15.060 ± 0.32	14.460 ± 0.42	15.460 ± 0.13
RBC (10^12^/L)	7.616 ± 0.20	7.428 ± 0.14	6.948 ± 0.20	7.402 ± 0.15
HCT (%)	43.900 ± 1.35	43.660 ± 1.54	40.340 ± 1.44	44.280 ± 0.37
RDW-SD	24.600 ± 0.46	26.600 ± 1.18	25.080 ± 0.59	25.460 ± 0.35
WBC (10^9^/L)	4.680 ± 0.56	5.660 ± 1.37	7.740 ± 0.74	6.600 ± 1.12
PLT (10^9^/L)	668.80 ± 47.18	675.80 ± 69.34	639.40 ± 89.86	605.80 ± 42.74

Biochemical parameters

Effect of Subacute Treatment of Rats With Jenacid® Herbal Product on Biochemical Parameters

Table 4 summarizes the clinical biochemistry test results of this study, AST and ALT, as well as total protein, plasma urea, and creatinine. Compared to the rats in the control group(G4), daily oral administration of JHP to all groups caused an increase in plasma AST and ALT, but the values remained in the normal range. There was a significant decrease in total protein levels in rats given 500 and 1000 mg/kg of JHP (P < 0.05). The urea and creatinine levels between the JHP-treated groups (250, 500, and 1000 mg/kg) and the control group after 28 days of treatment had an insignificant difference.

**Table 4:** Biochemical effects of subacute Jenacid® administration on liver and kidney function Presents the levels of liver enzymes (ALT, AST), total protein, urea, and creatinine, highlighting the impact of Jenacid® on liver and kidney function. Note: Each value represents the mean ± SEM for each group (n=5), against G4 (control). *P<0.05, AST: aspartate amino transferase, ALT: alanine amino transferase.

Parameter	G 1(JH_250_)	G 2(JH_500_)	G 3(JH_1000_)	G 4(Control)
LT	21.100 ± 1.60	22.400 ± 1.40	25.720 ± 2.04*	19.340 ± 0.39
AST	14.020 ± 1.24	18.200 ± 0.84	20.680 ± 1.76*	13.900 ± 0.21
Total protein	6.906 ± 0.22	6.294 ± 0.19*	5.884 ± 0.12*	7.832 ± 0.46
Urea	4.778 ± 0.56	5.372 ± 1.01	6.866 ± 0.60	5.380 ± 0.35
Creatinine	0.568 ± 0.04	0.812 ± 0.07	0.788 ± 0.06	0.556 ± 0.09

Anti-ulcer activity

Table 5 compares the number of ulcers, ulcer severity, ulcer index, and percentage of ulcer healing in rats treated with various doses of Jenacid® and omeprazole, providing evidence of Jenacid®’s ulcer-healing properties.

**Table 5:** Anti-ulcerative efficacy of Jenacid® in indomethacin-induced ulcers The results are presented as the means ± standard deviation (n = 5). *The mean difference is significant at P < 0.05, compared to positive control.

Groups	Dose	Average ulcer no.	Ulcer score	Ulcer index	% of ulcer healing
I	NC	5.2 ± 0.86	4.8 ± 0.20	10 ± 1.06	-
II	JHP_250_	3.4 ± 0.20	2.8 ± 0.58	6.2 ± 1.09	38%
III	JHP_500_	1.0 ± 0.32	1.1 ± 0.24	2.1 ± 0.56*	79%
IV	JHP_1000_	2.4 ± 0.40	1.4 ± 0.24	3.8 ± 0.65*	62%
V	Omez_20_	0.6 ± 0.24	0.8 ± 0.12	1.4 ± 0.37	86%

**Figure 1:**
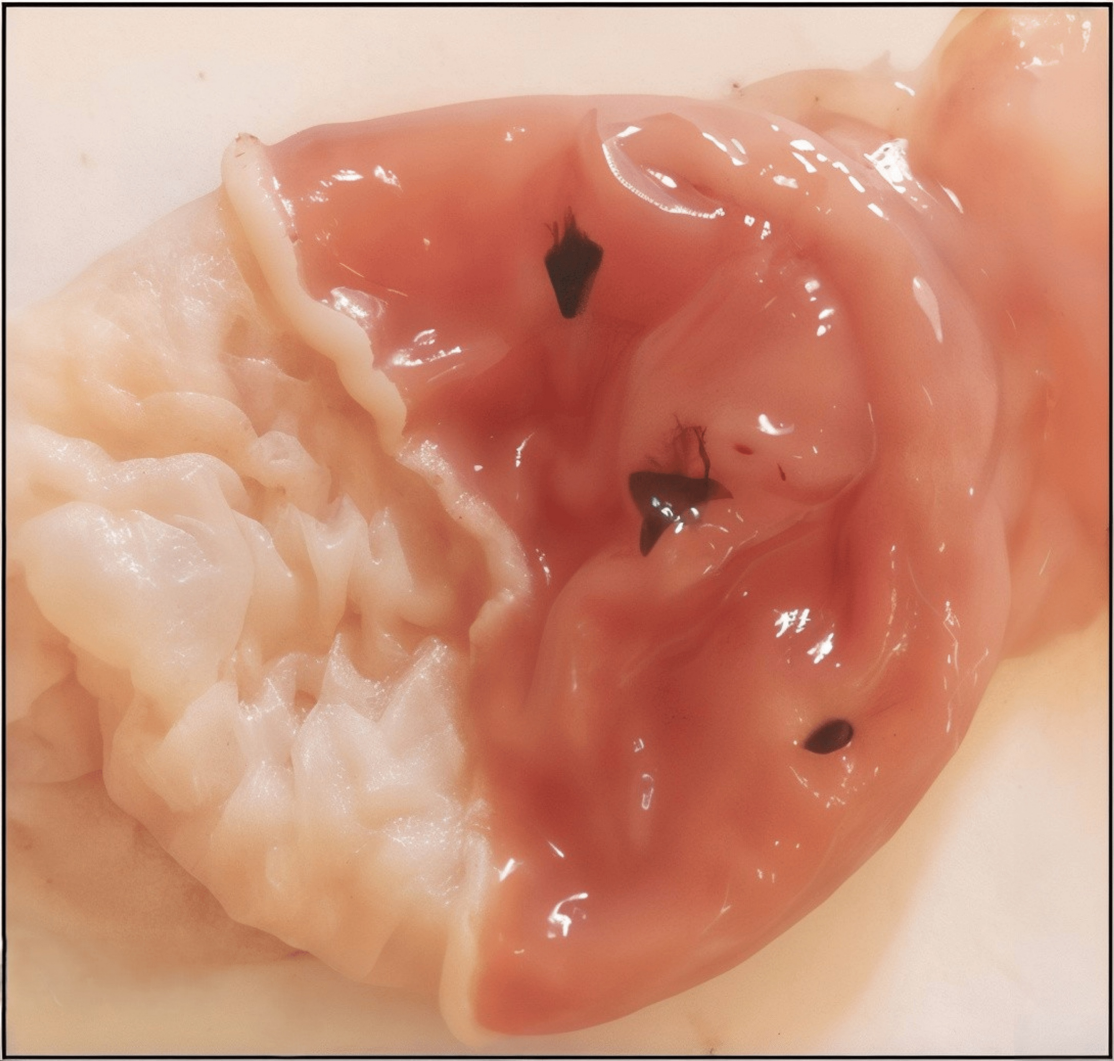
Gastric mucosa in the negative control group (distilled water) Image showing the extent of gastric damage in rats treated with distilled water after indomethacin-induced ulceration.

**Figure 2:**
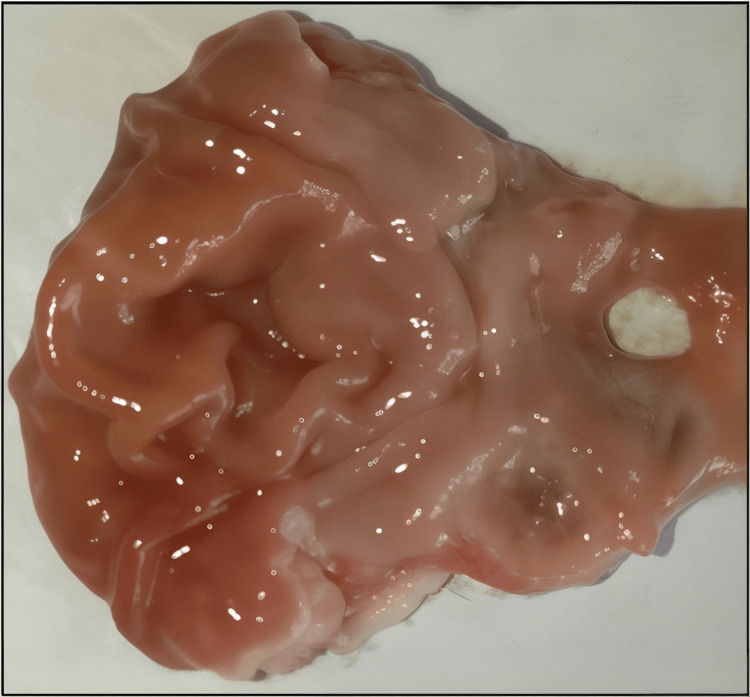
Protective effect of Jenacid® at 250 mg/kg on gastric mucosa Image depicting the partial healing of gastric ulcers in rats treated with Jenacid® 250 mg/kg, with noticeable reductions in mucosal damage.

**Figure 3:**
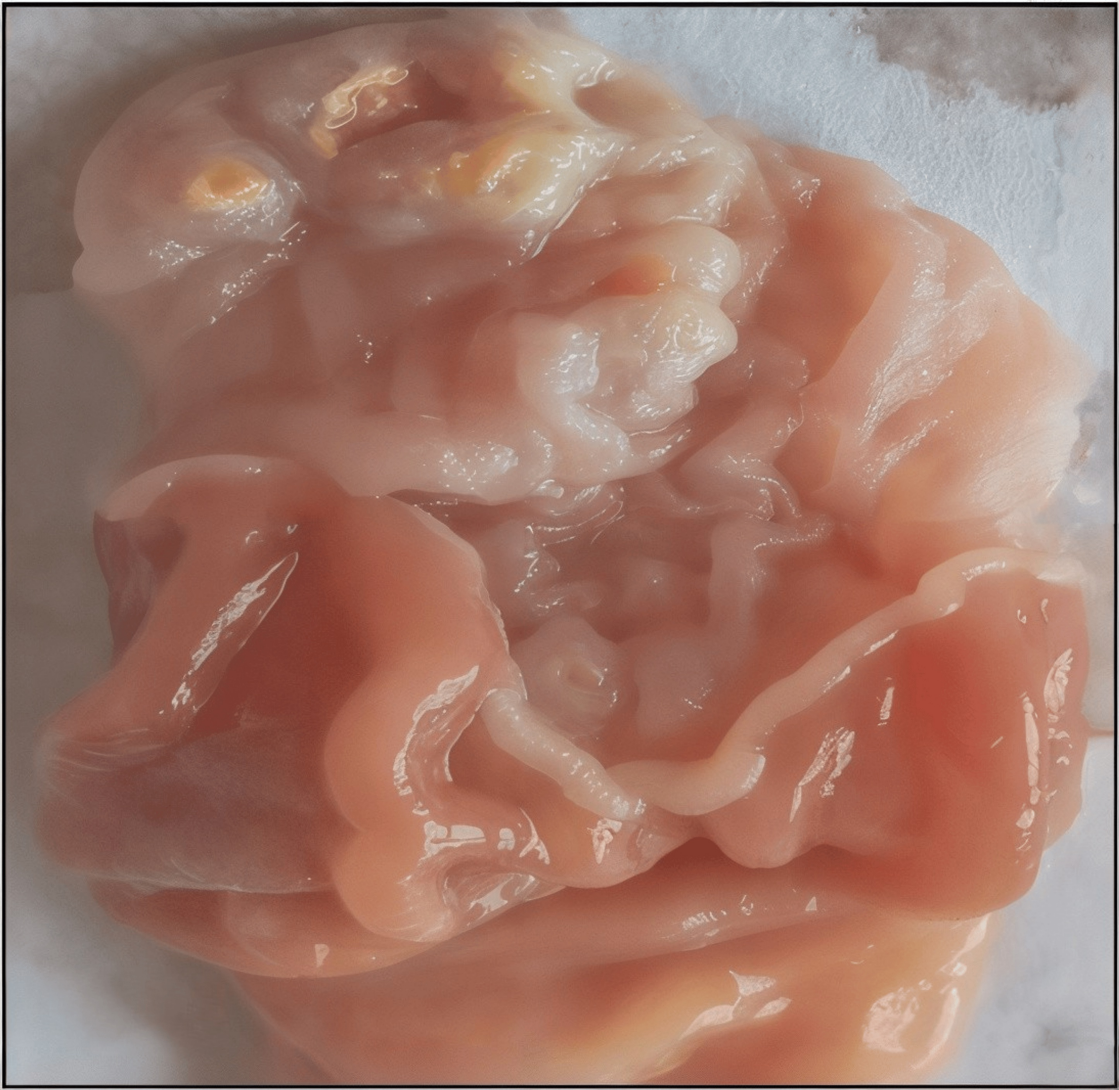
Ulcer healing properties of Jenacid® at 500 mg/kg Image showing substantial mucosal regeneration in the Jenacid® 500 mg/kg treatment group, indicating potent anti-ulcer activity.

**Figure 4:**
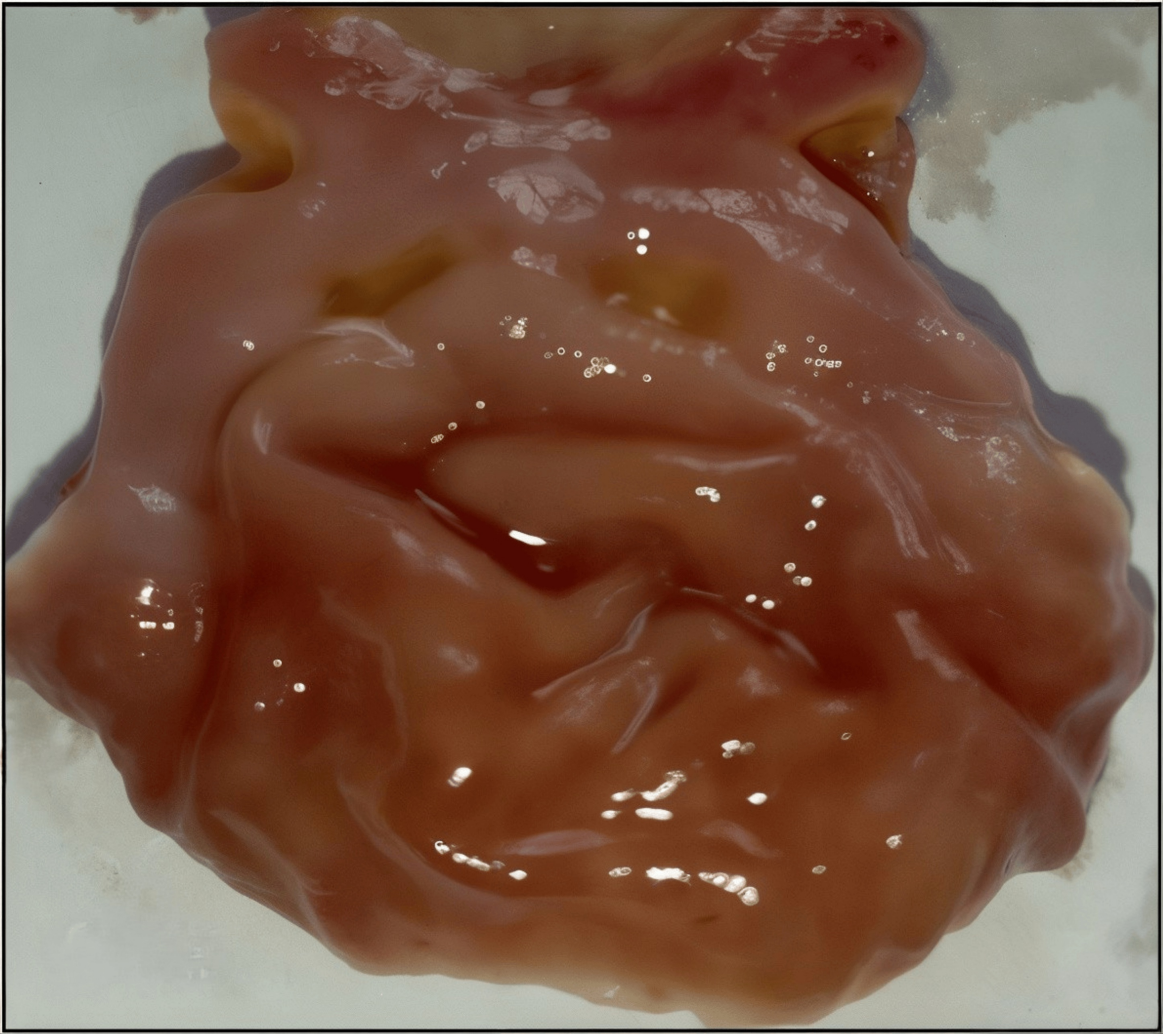
Enhanced ulcer healing by Jenacid® at 1000 mg/kg Image showing significant gastric mucosa recovery and reduced ulceration in rats treated with Jenacid® 1000 mg/kg.

**Figure 5:**
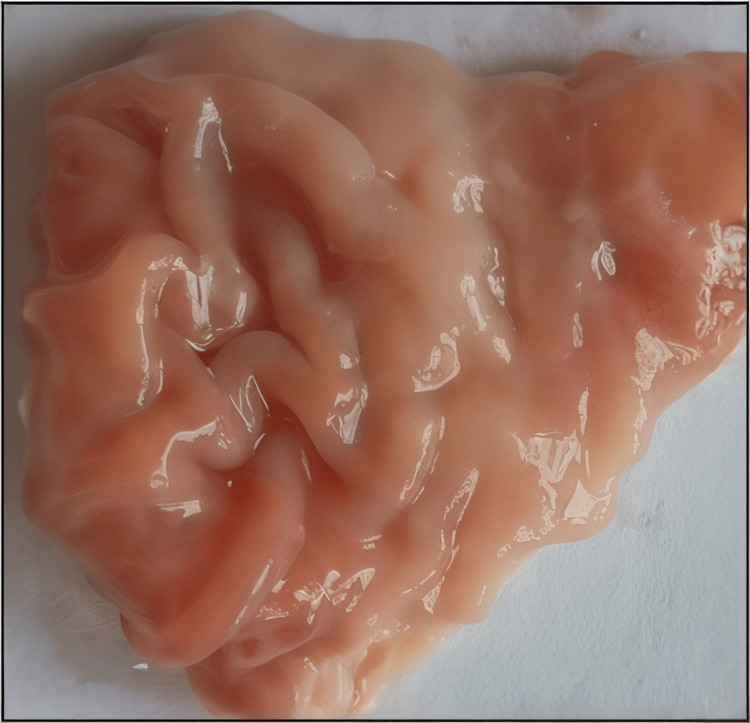
Standard ulcer treatment with omeprazole (20 mg/kg) Image showing the nearly complete healing of gastric ulcers in rats treated with omeprazole, serving as the standard reference for ulcer healing.

Figures 1-5 are the representative images of gastric mucosa from (1) the negative control group, (2) the Jenacid® 250 mg/kg group, (3) the Jenacid® 500 mg/kg group, (4) the Jenacid® 1000 mg/kg group, and (5) the omeprazole group. The negative control group shows significant mucosal erosion, while the Jenacid® 500 mg/kg and omeprazole groups demonstrate considerable mucosal protection with reduced damage.

## Discussion

The product was shown to be safe in rats up to the limit dose of 5000 mg/kg, and its median lethal dose (LD50) is greater than 5000 mg/kg. These results are in agreement with a study on the acute toxicity of *Warburgia ugandensis* [15] and a study on *Fagara zanthoxyloides*, which showed their acute toxicity to be above 5000 mg/kg [16]. The two medicinal plants are the ingredients in Jenacid® Herbal Product. Jenacid® herbal product was classified as reasonably safe on the Loomis and Hayes toxicity scale [17]. The safety of this product may be attributed to the low concentration of the phytochemicals or the presence of phytochemicals that counter the toxicity of others within the same preparation.

The product tested positive for flavonoids, saponins, tannins, sterols & triterpenoids, and alkaloids. These compounds were discovered in a similar investigation on *W. ugandensis* [18]. These secondary metabolites have antioxidant, anti-inflammatory, immune-stimulating, anti-cancer, and anti-ulcer properties [19]. Flavonoids are hypothesized to limit Helicobacter pylori growth, raise mucosal prostaglandin content, inhibit H+/K+-ATPase, and serve as free radical scavengers [20]. Saponins may activate mucous membrane protective factors, while tannins make the mucosa's outer layer less permeable to chemical irritants [21]. Furthermore, triterpenoids and alkaloid compounds have been shown to have substantial anti-ulcer action [22].

The results of repeated dose toxicity testing show target organ toxic effects and impacts on animal physiology, biochemistry, hematology profile, and histology. In this study, all animals were active and responded positively to stimuli during subacute exposure. There were no deaths or clinical symptoms of systemic or local adverse effects. A study on *Zanthoxylum chalybeum* subacute toxicity yielded similar results [23].

In general, an increase or reduction in an animal's body weight has been utilized as an indicator of a drug's detrimental effect [24]. The body weights of the treated rats did not differ substantially (P > 0.05) from the control groups in this investigation. This implies that the product has no influence on appetite or harmful effects on the animals' growth.

Previous research has demonstrated that hematological parameters are highly sensitive and can be utilized as accurate indicators of toxic chemical penetration [25]. In our study, the treated rats had no significant changes in WBC, RBC, HGB, or HCT at any dose (P < 0.05). As a result, no toxicological importance is assigned to these findings.

Renal and hepatic function are critical, with one being employed for waste product excretion and the other for intake metabolism, respectively [26]. It is necessary to know these two key organ physiological states in order to assess the toxicity of any new drug, which can be validated through biochemical estimation [27]. Renal and liver function tests were performed in this investigation. In addition, the protein profile and metabolic indicators were assessed. Clinical biochemistry signs related to liver disease include serum levels of liver enzymes (AST, ALT, and ALP) [25]. When compared to the control, serum levels of AST and ALT at all doses of JHP were statistically higher but still within normal ranges. The mean difference between group 3 (1000 mg/kg) and group 4 (control) was significant (P < 0.05). The total proteins in the JHP groups were significantly lower than the control groups, and this could be due to the presence of tannins, which bind proteins in the GIT. It is worth noting that the doses used were following OECD guidelines and not the human equivalent dose, which is much lower than what was used in the study; finding a suitable dose can be a limitation. Extended exposure periods (such as 90 days) may provide different results; however, since the ulcer treatment period indicated for Jenacid® Herbal Product is two to four weeks, longer-term studies may not be relevant.

## Conclusions

The acute and subacute studies, along with the observed anti-ulcer effects of Jenacid® herbal product administered orally, underscore the product's promising safety profile and efficacy in mitigating drug-induced peptic ulcers in Albino Wistar rats. The results from these preclinical trials suggest that Jenacid® herbal product effectively alleviates ulcer symptoms, presenting a viable alternative for ulcer management. However, while these findings are encouraging, they are limited to animal models and may not fully capture the product's effects in humans.

To validate these preliminary results and ascertain the therapeutic potential of Jenacid® herbal products in clinical settings, it is imperative to conduct rigorous clinical trials. These studies will help determine the product’s safety and efficacy in human subjects, establish appropriate dosing regimens, and explore potential side effects or interactions. Such clinical investigations are essential for translating the observed benefits from the animal model to practical applications in human healthcare.

## References

[1] Eichenseher J (2018). Peptic ulcer disease. Integrative Medicine.

[2] Archampong TN, Asmah RH, Richards CJ (2019). Gastro-duodenal disease in Africa: Literature review and clinical data from Accra, Ghana. World J Gastroenterol.

[3] Takezono Y, Joh T, Oshima T (2004). Role of prostaglandins in maintaining gastric mucus-cell permeability against acid exposure. J Lab Clin Med.

[4] Brzozowska I, Konturek PC, Brzozowski T (2002). Role of prostaglandins, nitric oxide, sensory nerves and gastrin in acceleration of ulcer healing by melatonin and its precursor, L-tryptophan. J Pineal Res.

[5] Awoussong P, Zaharia V, Ngameni B (2015). Synthesis, characterization, and anticancer activity of some thiazolic chalcones. Med Chem Res.

[6] Djimeli MN, Fodouop SP, Njateng GS, Fokunang C, Tala DS, Kengni F, Gatsing D (2017). Antibacterial activities and toxicological study of the aqueous extract from leaves of Alchornea cordifolia (Euphorbiaceae). BMC Complement Altern Med.

[7] Bhowmik D, Dubey P, Chandira M, Kumar KP (2009). A potential review on the role of herbal medicine in treating ulcer. Scholars Research Library.

[8] Kulkarani SK (2002). Handbook of Experimental Pharmacology.

[9] Kaur J, Kaur S, Mahajan A (2014). Herbal medicines: possible risks and benefits. SMU Med J.

[10] Adedapo AA, Falayi OO, Oyagbemi AA (2015). Evaluation of the analgesic, anti-inflammatory, anti-oxidant, phytochemical and toxicological properties of the methanolic leaf extract of commercially processed Moringa oleifera in some laboratory animals. J Basic Clin Physiol Pharmacol.

[11] Abeysena I Considering HCl resistant evaporator. J Chem Eng.

[12] Evans WC (1996). Pharmacognosy.

[13] Lorke D (1983). A new approach to practical acute toxicity testing. Arch Toxicol.

[14] Bhattacharya S, Chaudhuri SR, Chattopadhyay S, Bandyopadhyay SK (2007). Healing properties of some Indian medicinal plants against indomethacin-induced gastric ulceration of rats. J Clin Biochem Nutr.

[15] Karani LW, Tolo FM, Karanja SM (2013). Safety and efficacy of Prunus africana and Warburgia ugandensis against induced asthma in BALB/c mice. S Afr J Bot.

[16] Ogwal-Okeng JW, Obua C, Anokbonggo WW (2013). Acute toxicity effects of the methanolic extract of Fagara zanthoxyloides (Lam.) root bark. Afr J Pharm Pharmacol.

[17] Loomis TA, Hayes A (1996). Loomis's Essentials of Toxicology.

[18] Okello D, Komakech R, Matsabisa MG, Kang Y (2018). A review on the botanical aspects, phytochemical contents, and pharmacological activities of Warburgia ugandensis. J Med Plants Res.

[19] Mohod S, Bodhankar S (2011). Evaluation of antiulcer activity of the methanolic extract of Madhuca indica J.F. Gmel leaves in rats. Pharmacol J.

[20] Repetto MG, Llesuy SF (2002). Antioxidant properties of natural compounds used in popular medicine for gastric ulcers. Braz J Med Biol Res.

[21] Borrelli F, Izzo AA (2000). The plant kingdom as a source of anti-ulcer remedies. Phytother Res.

[22] Mitra P, Ghosh T, Mitra P (2015). Anti-gastric ulcer activity of Amaranthus spinosus Linn. leaves in aspirin-induced gastric ulcer in rats. SMU Med J.

[23] Engeu OP, Ralph T, Moses A (2008). Repeat-dose effects of Zanthoxylum chalybeum root bark extract: a traditional medicinal plant used for various diseases in Uganda. Afr J Pharm Pharmacol.

[24] Kharchoufa L, Bouhrim M, Bencheikh N (2020). Acute and subacute toxicity studies of the aqueous extract from Haloxylon scoparium Pomel by oral administration in rodents. J Ethnopharmacol.

[25] Fodouop SP, Tala SD, Keilah LP (2017). Effects of Vitellaria paradoxa (C.F. Gaertn.) aqueous leaf extract administration on Salmonella typhimurium-infected rats. BMC Complement Altern Med.

[26] Dramane P, Adama H, Yhi-pênê NJ (2019). Protective effect of bioactive fractions of C. Dalzielii against weight gain in mice fed with high-fat diet. Int J Recent Sci Res.

[27] Lager PS, Attema-de Jonge ME, Gorzeman MP, Kerkvliet LE, Franssen EJ (2018). Clinical value of drugs of abuse point of care testing in an emergency department setting. Toxicol Rep.

